# Comparative Analysis of Social Support in Online Health Communities Using a Word Co-Occurrence Network Analysis Approach

**DOI:** 10.3390/e24020174

**Published:** 2022-01-25

**Authors:** Mengque Liu, Xia Zou, Jiyin Chen, Shuangge Ma

**Affiliations:** 1 School of Journalism and New Media, Xi’an Jiaotong University, No.28 Xianning West Road, Xi’an 710049, China; mengqueliu@xjtu.edu.cn (M.L.); zx124557896@xjtu.edu.cn (X.Z.); cjyxajd@mail.xjtu.edu.cn (J.C.); 2 Department of Biostatistics, Yale University, 60 College Street, New Haven, CT 06520, USA

**Keywords:** online health community, social support, network analysis, cancer

## Abstract

Online health communities (OHCs) have become a major source of social support for people with health problems. Members of OHCs interact online with others facing similar health problems and receive multiple types of social support, including but not limited to informational support, emotional support, and companionship. The aim of this study is to examine the differences in social support communication among people with different types of cancers. A novel approach is developed to better understand the types of social support embedded in OHC posts. Our approach, based on the word co-occurrence network analysis, preserves the semantic structures of the texts. Information extraction from the semantic structures is supported by the interplay of quantitative and qualitative analyses of the network structures. Our analysis shows that significant differences in social support exist across cancer types, and evidence for the differences across diseases in terms of communication preferences and language use is also identified. Overall, this study can establish a new venue for extracting and analyzing information, so as to inform social support for clinical care.

## 1. Introduction

A cancer diagnosis and treatment can cause significant changes to a person’s path in life and affect his/her daily activities, work, relationships, and family roles. Cancer patients (and their surrounding members) often suffer from a high level of psychological stress, which can lead to anxiety and depression. They strongly demand social support, which is broadly defined as resources or aids that are exchanged by members within a specific community. Extensive research [1,2,3] has reported social support as a complex construction with direct and buffering effects on a person’s well-being and psychological adjustment to cancer. For example, studies have suggested the association between social support and cancer progression [4]. In addition, insufficient social support can lead to poor health behaviors, which may result in an increased vulnerability toward cancer and its associated mortality [5]. It has also been identified as a consistent indicator for survival.

According to the Health Information National Trends Survey, the proportion of cancer survivors reporting internet use has increased over time, from 49.5% in 2003 to 76.9% in 2017 [6]. Consistent with that, social support is also increasingly exchanged via computer-mediated communication, which has been referred to as computer-mediated social support. It can be developed among strangers whose only connection is their common affliction or concern about a source of personal discomfort. The anonymous nature of online communities also allows patients to exchange personal concerns and advice without the fear of being judged or recognized [7]. We refer to published studies for more discussions on the advantages of computer-mediated social support [8,9,10]. Online health communities (OHCs) are online social networks with a focus on health. OHCs can be categorized as either general-purpose communities or those dedicated to a specific health issue. Many OHCs have their own websites, while others are built on existing social networking services, such as Facebook. Compared to traditional health-related websites that only allow users to retrieve information, OHCs can increase members’ ability to interact with peers facing similar health problems and, as a result, better meet their immediate needs for social support. People show emotional support for others in OHCs by offering encouragement, reassurance, compassion, etc. OHCs are helpful in empowering patients through personal participation and providing access to information as well as emotional support.

Understanding how members of these online groups interact with each other and make use of online support resources is of critical interest. A handful of content analyses have been conducted, examining the nature of support messages communicated in OHCs [11]. In several studies that analyzed a variety of cancer support groups, information support was found to be the predominant type of support exchanged [12,13]. Some other studies reported that emotional support was the most frequent type of support message [14,15]. Questions, though, about when and why social support messages in computer-mediated contexts vary systematically remain largely unanswered [16]. Blank et al. [17] and Seale et al. [18] revealed significant gender differences. There is also evidence that the support needs of those who were diagnosed, and their families, vary by disease [12,19,20]. It is noted that these studies are mostly limited to breast cancer and prostate cancer, which are mostly gender-specific. Our literature review suggests that, in general, differences across diseases have not been sufficiently examined—something that is critical for understanding patients’ needs related to information, emotional support, and relationship-building in OHCs. Only by understanding patients’ more specific perceptions and needs can we further optimize the designs and services of OHCs, especially for cancer survivors, who have complex support needs and require different levels of care [21].

Our objective is to provide a detailed and inductively generated account of cancer-type differences in a large number of postings in online cancer support forums. To this end, a novel approach is applied to better understand the types of social support embedded in OHC posts. Different from some previous studies that relied on a commensurate coding scheme with all posts coded [22], which is not feasible with a large amount of data, our approach, based on a word co-occurrence network analysis technique, can provide a macroscopic field-wide view to extract information from big data, making it possible to process a massive amount of online community data. Some other studies adopted quantitative analysis approaches. For example, Seale et al. [18] conducted a comparative keyword analysis to facilitate an interpretive and qualitative examination focused on the meanings of word clusters associated with keywords. There are limitations, however, such as a lack of relevance of word clusters and an inaccurate expression of text themes. Wang et al. [23] used machine learning techniques to reveal the types of social support embedded in each post of an OHC. Wu et al. [24] proposed a social support classification method, using an LDA (linear discriminant analysis) to extract topic features from data. A significant limitation of this analysis is that a certain amount of human annotation is needed, which can be time-consuming and subjective. In addition, an unbalanced data distribution can affect the accuracy of prediction and performance. In this study, the adopted analysis approach can advance from the aforementioned and other studies and directly overcome their limitations. Text data are organized and analyzed with a network perspective, which is system-oriented. Our analysis can identify patterns and relationships among all the words in a system. It can capture properties of individual words and provide insight on how individual words are tied to a larger web (collection of interconnections).

Overall, this study fits well in the scope of information theory-based research. Specifically, it extracts information by conducting complex text mining, and generates knowledge on a complex system by conducting an advanced network analysis, which can more effectively describe variables by taking a system perspective and modeling interconnections. Although the analytic methods adopted in this article have roots in the existing literature, their “combination” and application to a new domain and new biomedical questions are novel. The most essential merit of this study may come from its data analysis findings, which can reveal the social support needed for multiple deadly cancers and the significant differences across cancer types: this has been suggested in the literature but not well quantified to date. The findings can be valuable for stakeholders at multiple levels including healthcare providers, patients, family members, and others. This study can also serve as a prototype for future social support analyses using state-of-the-art network and information analysis techniques, and noting that the existing social support analysis has mostly been based on less advanced methods.

## 2. Materials and Methods

### 2.1. Data Source

Patientslikeme.com (PLM) is the world’s largest personalized health network, with a growing community of more than 830,000 users. It was designed to facilitate information-sharing between users within disease-specific communities, with the goal of improving the well-being of all users through knowledge derived from shared, real-world experiences and outcomes. In addition to general social networking service (SNS) tools such as user profiles, comments, and private messages, each community has disease-specific tools that allow patients to track and share relevant information such as symptoms, treatments, and medical data. These features have enabled PLM to play a leading role in empowering patients and facilitating social support exchanges and communication online. We note that PLM is not specific to cancer. However, it may still be one of the best resources for studying cancer social support. Beyond the aforementioned advantages, it also has a close working relationship with various healthcare providers. For example, two-thirds of its users felt that their healthcare providers approved/supported using PLM, and about one-third had printed out their patient profiles for use during healthcare visits [25].

PLM has a representative cancer community of more than 50,000 people with over 50 types of cancers, and it is focused on providing customized, disease-specific services that are closely related to our research goal. Extensive research into patient perspectives has been based on this information source. For example, there have been several evaluations of patient perspectives on diseases as well as patient-reported clinical and treatment experience studies of social support groups [26,27]. Other OHCs, such as Breastcancer.org [28], Google Groups [19], and WebMD [29], have also been utilized as data resources in related research.

**Figure 1:**
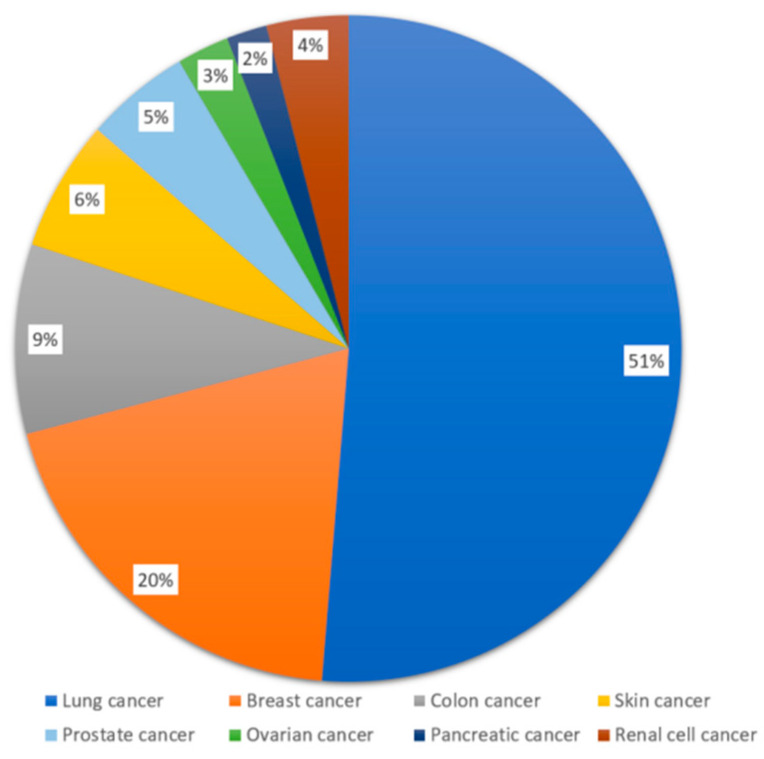
Percentages of posts for the eight types of cancer.

A web crawler was designed and used to collect data from the PLM online cancer forums, which were launched in 2011. The original dataset consists of all the public posts and user profile information from February 2011 to September 2020. There are 12,150 posts that were contributed by 1358 users who were cancer patients or family members. All posts were in English. The cancer patients were then filtered (according to tags and conditions), leading to 6262 posts. Most of the posts (87.85%) are related to eight cancers. Our exploration shows that the dominating majority of patients had a single type of cancer, which matches clinical practice. Additional details are presented in Figure 1. Our study is centered around these eight specific cancers.

### 2.2. Method and Procedures

The key steps include the construction of the word co-occurrence network, module detection, social support examination, and interpretation. They are discussed in detail in the following subsections.

**Step1** **: Word Co-Occurrence Network Construction**

The posts are split into sentences. For pre-processing, we first conduct tokenization. Stop words that are not informative are removed. Punctuation marks are excluded. Multi-word tokenization is also conducted to expand a raw token into multiple syntactic words. A word co-occurrence network is created with unigram tokens and concatenated multi-word units.

**Figure 2:**
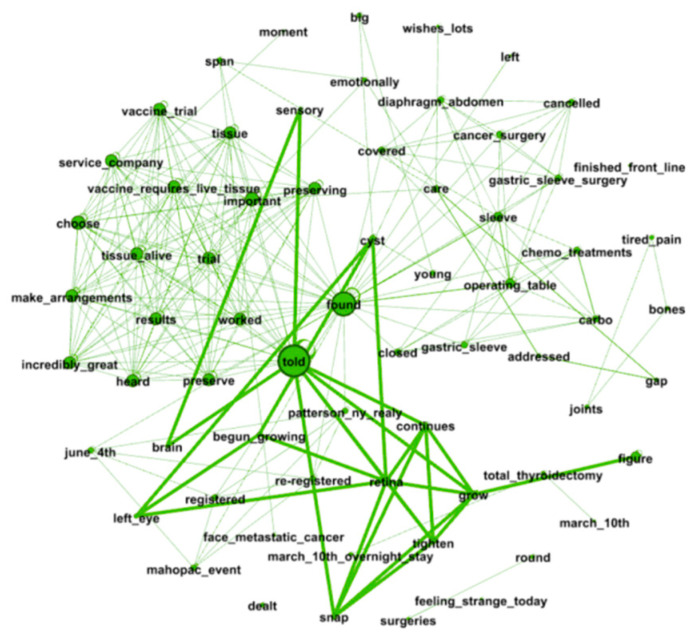
A sample word co-occurrence network for randomly selected posts.

A word co-occurrence network can be expressed as G=(V, E), where V is a set of nodes (where each node represents a word) and *E* is a set of edges. Edge $e_{ij}\in E$ connects nodes i and j if those two words co-occur within at least one sentence. The number of edges is denoted as m=|E|, and n=|V| denotes the number of nodes. The degree of a node i is the number of edges connected to that node, that is, $k_{i}=|\;\{j\;\in \;V\;|\left\{i,\;j\right\}\in \;E\}|$. The weight $w_{ij}$ of edge $e_{ij}$ is defined as the count of joint word occurrence, describing the co-occurrence relationship between the corresponding words in one sentence. The network is undirected by construction. Figure 2 shows a representative word co-occurrence network plotted using the software *Gephi* and containing information on the words and semantic structures. Some important statistical parameters that characterize a network are examined. First, the average shortest-path length (*ASPL*) is the average value of the shortest-path length between any two nodes in the network, which is calculated as:

$$ASPL=\frac{2\sum_{i>j}d_{ij}}{n\left(n-1\right)},$$

where $d_{ij}\;$ is the shortest-path length between nodes *i* and *j*. Second, the clustering coefficient of the network CC is the average of the clustering coefficients of all the nodes in the network defined as:

$$CC=\frac{1}{n}\sum_{i}\frac{m_{i}}{k_{i}\left(k_{i}-1\right)/2},$$

where $k_{i}$ is the degree of node i, and $m_{i}$ is the number of edges among the $k_{i}$ neighbor nodes. For example, for an Erdös–Renyi random network, its average shortest-path length is $ASPL_{r}\approx \;\ln\left(n\right)/\left(\ln\left(2m\right)-\ln\left(n\right)\right)$, and its clustering coefficient is $CC_{r}\approx \;2m/n\left(n-1\right)$. A network is said to be a small-world network if $ASPL\approx ASPL_{r}$ and $CC\approx CC_{r}$ [30]. Third, degree distribution p(k) is defined as the probability that a randomly chosen node has exactly degree k. For example, if p(k) satisfies the power-law degree distribution, that is, $p\left(k\right)\;\propto k^{-\gamma }$, where γ is a positive constant, then the network is said to be scale-free [31].

The study of co-occurrence can allow researchers to quantitatively describe the semantic structures of posts. However, significant challenges appear immediately. The word co-occurrence network of posts is usually very hard to visualize, and it is impossible to directly extract meaningful information. As such, there is a strong need to simplify the network, which can reduce complexity, improve visualization, and serve other purposes. One approach is to construct subgraphs, in which most of the useful information contained in the initial graph can be preserved. Here, we achieve this goal via network modules.

**Step2: Module Detection**

A module is defined as a set of densely connected nodes that are sparsely connected to the other modules in the network. The Louvain algorithm [32], which is based on the optimization of the quality function known as modularity over all possible divisions of a network, is adopted in this analysis and realized using the *Gephi* software. More specifically, this algorithm identifies modules by minimizing:

$$Q\left(c\right)=\frac{1}{2M}\underset{i}{\sum }\underset{j}{\sum }\left[w_{ij}-\lambda \frac{\ell_{i}\ell_{j}}{2M}\right]\delta_{ij}\left(c\right),$$

where c is a partition of nodes, $w_{ij}$ is the edge weight between nodes i and j, λ is a tuning parameter,

$$M=\frac{1}{2}\underset{i}{\sum }\underset{j}{\sum }w_{ij},$$

$$\ell_{i}=\underset{j}{\sum }w_{ij},$$

and

$$\delta_{ij}\left(c\right)=\{\begin{array}{cc} 1 & if\;\;c\left(i\right)=c\left(j\right)\\ 0 & otherwise \end{array}.$$

Here c(i) denotes the module to which node *i* belongs in the partition c.

The algorithm can unfold a complete hierarchical modular structure for the network, thereby giving access to different resolutions of module detection. In *Gephi*, the resolution parameter, which describes how much between-group edges impact the modularity score, determines the granularity level at which modules are detected [33], with a low-resolution value resulting in more modules. It has been suggested that this algorithm outperforms all other module detection methods in computation time. Moreover, highly satisfied module detection has been observed in practice. For our analysis, module detection of the word co-occurrence network can reduce the size of data, and the analysis of co-occurrences in an individual module can allow researchers to keep track of the semantic structures, which are useful in understanding social support.

**Step3: Social Support Quantification and Interpretation**

**Figure 3:**
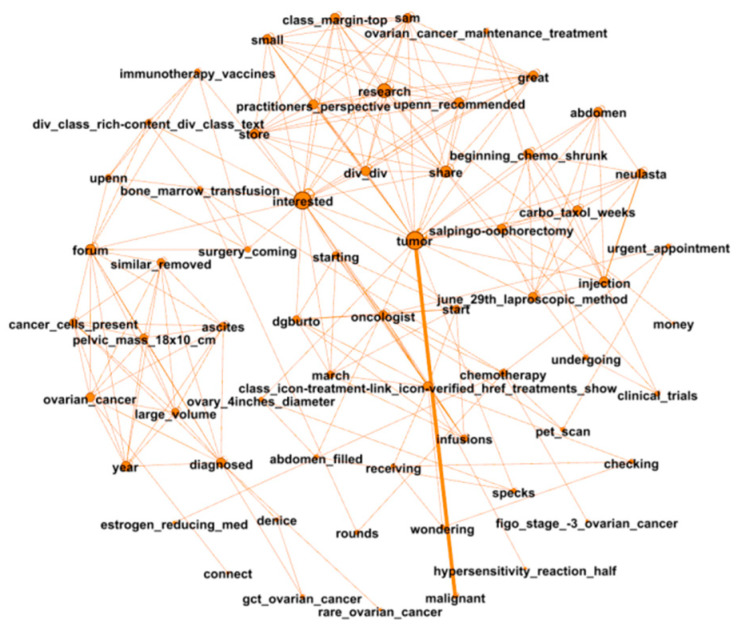
A sample module from the word co-occurrence network for ovarian cancer.

The analysis of word co-occurrences involves clustering words together without breaking their semantic links. In this step, we examine social support by analyzing the semantic structures of the identified modules. As a representative example, Figure 3 presents a module in the word co-occurrence network for ovarian cancer. The words grouped in one module are likely to describe tightly connected topics. For example, most of the words in Figure 3 are related to treatments and medical terminologies. As such, this module can be considered as describing informational support.

- The Taxonomy of Social Support.

Several taxonomies have been developed for the categories of support messages (see for example, [34,35]). Literature on social support suggests that OHCs mainly offer three types of social support: informational support, emotional support, and companionship [11,36]. Informational support is the transmission of facts, suggestions, and/or guidance to community users. Example topics include medication side effects, ways to deal with a symptom, experience with a physician, and medical insurance problems. Emotional support is the expression of understanding, encouragement, empathy, affection, affirmation, caring, and concern. Such support can help reduce stress and anxiety. Companionship consists of chatting, humor, teasing, and discussions of daily life that are not necessarily related to health problems. Examples include diet plans, birthday wishes, holiday plans, and online scrabble games. Companionship helps expand or reinforce a group member’s connections.

Through the quantitative analysis of semantic structures, the prevalence of specific types of support messages can be revealed. To do this, the first step is to calculate the proportion of edges in each module, which is defined as:

$$P_{C_{k}}=\frac{\sum_{i\;\in C_{k}}\{j\;\in C_{k}|\left\{i,j\right\}\in E\}}{\sum_{k=1}^{K}\sum_{i\;\in C_{k}}\{j\;\in C_{k}|\left\{i,j\right\}\in E\}},\;\;k=1,\ldots ,K,$$

where K is the number of modules, $C_{k}$ represents module k, $\underset{i\;\in C_{k}}{\sum }\{j\;\in C_{k}|\left\{i,j\right\}\in E\}$ denotes the sum of edges between nodes in $C_{k}$. Then, we can compute the proportion of each social support category by summing up the proportions from the individual modules. Exploring communication preferences and language use can also be achieved by taking a closer look at the semantic structures.

## 3. Results

We apply the analysis approach described above to the data on individual cancers. Pancreatic cancer is highlighted as a representative example.

### 3.1. Word Co-Occurrence Network

Sentences drawn from the posts were tokened prior to the co-occurrence search, resulting in a list of unique co-occurrence pairs. The word co-occurrence network was then constructed for each cancer. Summary information on the word co-occurrence networks is provided in Table 1. Based on this, an overview of the co-occurrence networks can be provided.

Compared to a same-scale random network, all the networks have similar average shortest-path lengths and higher clustering coefficients. For example, the average shortest-path length of the pancreatic cancer network is 3.595 (in comparison, an Erdös–Renyi random network has a value of 2.258), and the average clustering coefficient is 0.861 (in comparison, an Erdös–Renyi random network has a value of 0.013). This suggests the presence of the small-world phenomenon in the networks.

In the analysis of degree distribution, it is found that all networks exhibit power-law degree distributions, with the power-law exponent γ ranging between 2.4 and 4.8. Table 1 shows that γ of the ovarian cancer network is the largest, and that of the lung cancer network is the smallest. The scale-free characteristics suggest that the connectivity values of a small number of nodes are quite large (with a large number of connections), rendering them leading roles in the networks. On the other hand, most other nodes have limited connections.

### 3.2. Module Detection

**Figure 4:**
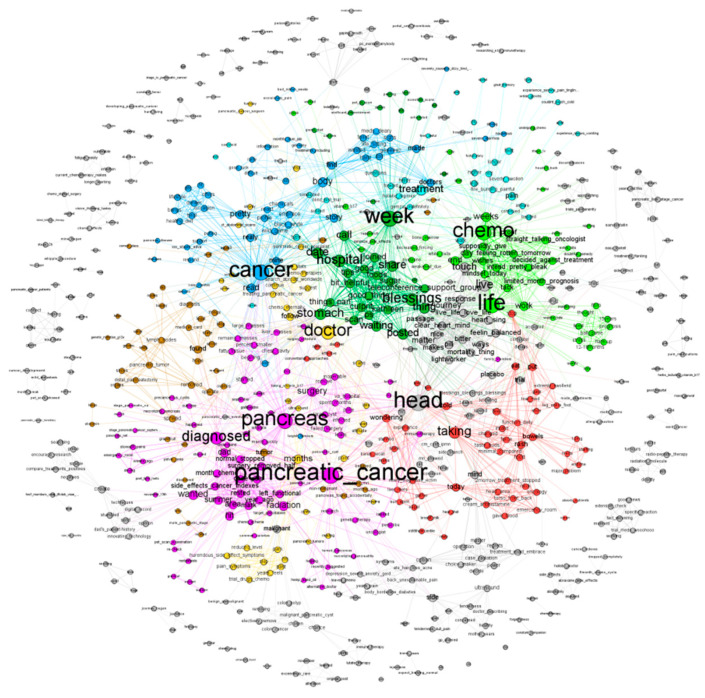
Word co-occurrence network for pancreatic cancer. Different modules are represented using different colors.

Take pancreatic cancer as an example. When we visualize its network (Figure 4), words in different modules are represented with different colors. Under the default resolution value of 1.0, there are 72 modules, and the modularity is 0.769. Modules with fewer than five words are removed to improve presentation, leading to 25 modules. Among the remaining modules, the average clustering coefficient is 0.890, suggesting a significant clustering effect. The silhouette for each module is also calculated. The mean silhouette value is 0.649. The silhouette values of the five largest modules are shown in Table 2, which suggest a satisfactory partitioning of the network. The same analysis is also conducted on the other cancers, and the summary of the module detection results is presented in Table 3.

### 3.3. Social Support Quantification and Interpretation

Summary information for the five largest modules for pancreatic cancer is shown in Table 2. It is observed that the themes of modules 1–4 are mainly concentrated around cancer information, that is, information social support. The keywords of module 5 are mostly associated with the feelings of patients, corresponding to emotional social support. With a similar analysis of the other modules, the proportion of edges in each module is calculated, and the proportions of different social support types after aggregation are obtained. Results are shown in Table 4.

#### 3.3.1. Differences across Diseases in Types of Social Support

Table 4 shows the proportion of each social support category for each cancer type. Overall, information support (mean 47.14%) and companionship (mean 28.26%) are exchanged most frequently. Sharing is caring, and most posts talk about medical treatments and daily life. The Chi-squared analysis confirms that the overall distribution of social support categories is significantly different across cancer types (*p* < 0.001). Specifically, lung cancer, colon cancer, and pancreatic cancer have the highest percentages (above 50%) of information support. Ovarian and breast cancers have the lowest percentages of information support. Breast cancer has the highest percentage of emotional support (40.45%), followed by prostate cancer (36.73%), ovarian cancer (36.43%), and skin cancer (24.19%). Skin cancer has the highest percentage of companionship (33.79%), while breast cancer (18.87%) and prostate cancer (22.12%) have the lowest.

#### 3.3.2. Differences across Diseases in Communication Preference and Language Use

**Figure 5:**
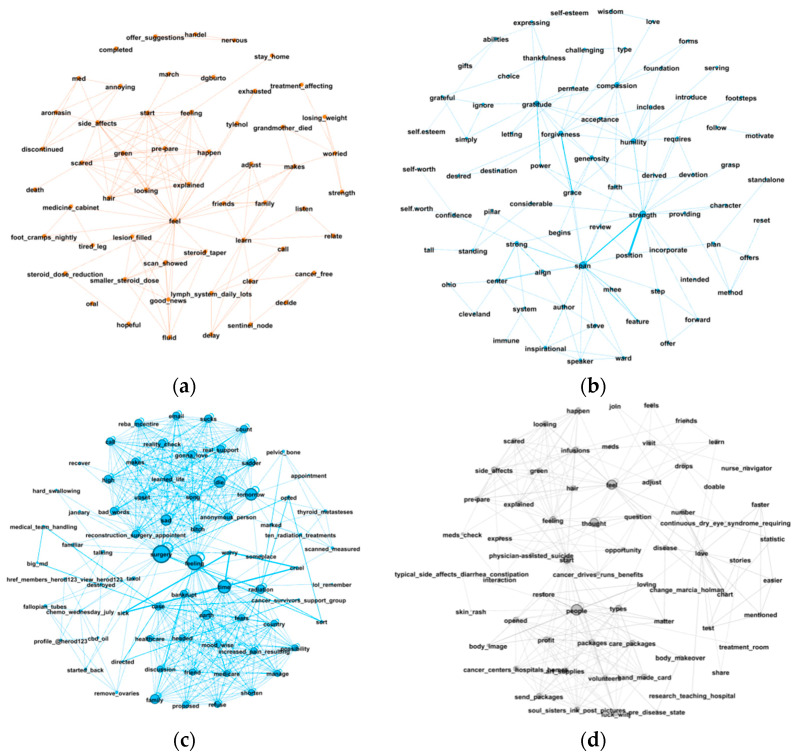
Emotional social support revealed by network modules: (**a**) breast cancer; (**b**) prostate cancer; (**c**) ovarian cancer; (**d**) skin cancer.

**Figure 6:**
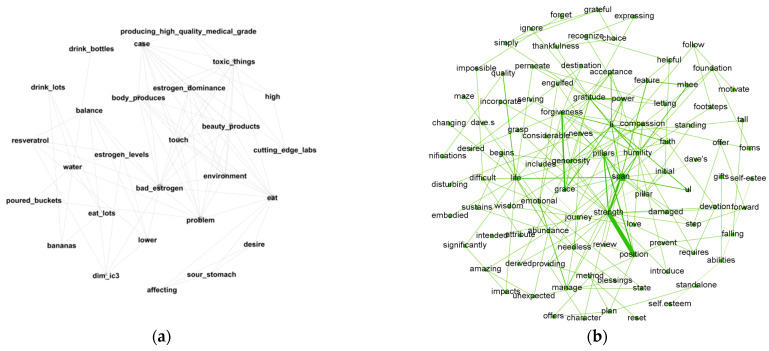

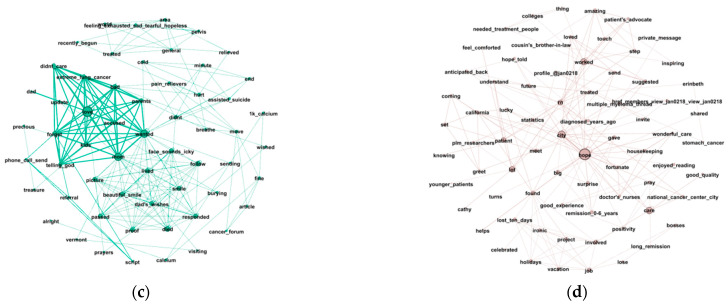
Companionship revealed by network modules: (**a**) breast cancer; (**b**) prostate cancer; (**c**) ovarian cancer; (**d**) skin cancer.

There is evidence of differences in language use and communication preference across diseases. Four cancers (breast, ovarian, prostate, and skin) have pronounced communication preference and language use patterns. Figure 5 shows the representative network modules, revealing the emotional support of these four cancers. It is observed that breast and ovarian cancer patients mainly talked about their pains and feelings, and their language style was sentimental. In comparison, prostate cancer patients talked more about their thoughts and beliefs, and their language style was calmer and more rational. Figure 6 shows the companionship traits of the four cancers. Skin and breast cancer patients mainly talked about their daily lives, ovarian cancer patients talked more about their family members, and prostate cancer patients talked more broadly. Differences in language use and communication preference mainly exist in the categories of emotional support and companionship. Overall, these findings can reveal several key differences in the use of OHCs across cancer types.

## 4. Discussion

Our findings are mostly consistent with published research. For example, information support has been identified as the most common type of social support, and published literature has suggested that messages of emotional well-being and medical-related comments are most common on breast cancer sites [17,19,37]. Meanwhile, our research has also added to the existing knowledge of the significant differences between social support categories across cancer types. For example, lung cancer, colon cancer, and pancreatic cancer survivors have been found to mainly utilize OHCs for information-gathering. Notably, prostate cancer survivors also used OHCs as a source of emotional support. Breast, ovarian, prostate, and skin cancer survivors appeared to be in most need of emotional social support. This is likely because people with these cancers had to bear more mental pressure and had a higher risk of also experiencing depression after a new cancer diagnosis [38]. For skin cancer, the high percentage of companionship indicates that the survivors had many daily struggles that led them to seek out support.

Besides adding to existing knowledge by complementing and extending previous research into computer-mediated social support communicated by cancer patients, our analysis has also demonstrated the need for greater recognition of the differences between people with different types of cancer. This knowledge can assist in the design of OHCs. The work can also be a resource for guiding cancer survivors and their families to OHCs that tend to focus more on their specific types of cancer and issues. Similarly, clinicians need to be more aware of the different needs of patients and their families and be able to direct them to online resources that are the most likely to be supportive. In this line, recent studies have shown that the internet has changed the patterns of doctor–patient communication. Social support in OHCs has sometimes played an ambiguous role, making patients behave in a strategic, uncooperative way toward physicians [39,40]. Patient care services have been recommended to enhance the patient–physician relationship. More studies on patients’ specific support needs and patient–physician cooperation are needed.

The adopted analysis method can also be used, along with or in replacement of machine learning techniques, in the identification of user roles in OHCs. Further studies on user roles (for example, the differences between lurkers and posters, their specific behaviors, and impact) are also warranted.

### Limitations

This study inevitably has limitations. Although PLM is representative and its data has also been examined in other published studies, it is a single OHC and may have a problem of biasedness; although, this has not been observed in existing studies. We have extracted all cancer forum data from PLM. Still, the amount of data for some cancers is limited. This may be true for pancreatic, ovarian, and renal cell cancers. Another data limitation is the possible lack of reliability. Medical information researchers have found that social media sites are identified by limited information [41]. Online users may also be vulnerable to both hidden and overt conflicts of interest, and so they may be incapable of interpreting [42]. In this dataset, there is a lack of information on the duration of diagnosis. As such, we are not able to conduct, for example, a longitudinal analysis to examine temporal trends. Another missed opportunity is that, with a small number of patients with multiple types of cancers, we are not able to provide insights into poly chronic conditions.

There may also be methodological limitations. For example, there is an emphasis on a module-based analysis over individual-message based, which may lead to certain challenges in result interpretation. We have studied the most essential network properties, and it may be of interest to explore more subtle network information.

## 5. Conclusions

This study has made both domain-specific and methodological contributions to the investigation of OHC use among cancer survivors. There is evidence, some of which confirms and some of which adds to the existing literature, about the significant differences across diseases in terms of social support needs. Specifically, lung cancer, colon cancer, and pancreatic cancer survivors mainly utilized OHCs to meet information support needs. Healthcare providers and physicians are recommended to provide guidance to patients and families on how to gather information and verify its authenticity. Breast, ovarian, prostate, and skin cancer survivors were found to be the most in need of emotional support. For them, targeted patient care can be advice and help to build healthy relationships in a community. Moreover, there is evidence for differences across diseases in language use and communication preference when exchanging social support. For example, skin and breast cancer patients mainly talked about their daily lives, ovarian cancer patients talked more about their family members, and prostate cancer patients talked more about their thoughts and beliefs. Getting familiar with patients’ communication preferences can be valuable for establishing the patient–provider bond. With collaboration, liking, and trust, patients are more likely to adhere to treatment especially for long-term medical issues. This work has also introduced a novel method for social support quantification and interpretation, which has multiple advantages over the analyses applied in previous studies.

## Figures and Tables

**Table 1:** Summary of the word co-occurrence networks.

Cancer Type	Sentences	Words	Co-Occurrence Pairs	$ASPL/ASPL_{r}\;$	$CC/CC_{r}\;$	γ
Lung cancer	15,690	12,830	196,620	3.167/2.764	0.789/0.001	2.416
Breast cancer	3222	4059	48,559	3.481/2.617	0.821/0.003	2.930
Colon cancer	2746	3524	57,430	3.188/2.344	0.826/0.005	3.017
Basal cell skin cancer	1295	1462	12,124	3.901/2.595	0.831/0.006	3.453
Prostate cancer	751	2005	28,475	3.409/2.272	0.867/0.007	3.056
Ovarian cancer	585	936	10,842	3.592/2.177	0.884/0.012	4.802
Pancreatic cancer	315	729	6749	3.595/2.258	0.861/0.013	3.939
Renal cell cancer	848	1196	9692	4.054/2.544	0.858/0.007	3.414

**Table 2:** Information on the five largest modules for pancreatic cancer.

Module ID	% of Edges	Silhouette	Selected Keywords
1	11.35%	0.503	pancreatic cancer; diagnosed; side effects; surgery; left functional
2	7.90%	0.546	chemo; life; oncologist; monitoring; weeks
3	6.32%	0.615	cancer; healthy diet; drinker; fatty tissue; chest cavity
4	6.08%	0.771	doctors; medical cars; scans; sign; pain symptoms
5	4.39%	0.791	treatment; pain; happy; awful; painful

**Table 3:** Summary of module detection.

Cancer Type	Modularity	*CC*	Silhouette	Number of Modules
Lung cancer	0.414	0.855	0.426	28
Breast cancer	0.646	0.842	0.433	29
Colon cancer	0.576	0.840	0.506	27
Basal cell skin cancer	0.751	0.857	0.575	29
Prostate cancer	0.685	0.898	0.802	28
Ovarian cancer	0.786	0.903	0.593	27
Pancreatic cancer	0.769	0.890	0.649	25
Renal cell cancer	0.797	0.877	0.503	26

**Table 4:** Proportions of different social support categories.

Cancer Type	Information Support	Emotional Support	Companionship
Lung cancer	54.94%	13.32%	31.74%
Breast cancer	40.68%	40.45%	18.87%
Colon cancer	58.81%	8.99%	32.20%
Basal cell skin cancer	42.02%	24.19%	33.79%
Prostate cancer	41.15%	36.73%	22.12%
Ovarian cancer	37.22%	36.43%	26.35%
Pancreatic cancer	54.34%	13.13%	32.53%
Renal cell cancer	47.92%	23.61%	28.47%

## Data Availability

The analyzed data are in the public domain and accessible to all researchers. However, we do not have the authority to re-distribute data.
